# Artificial intelligence for perioperative precision in surgical oncology

**DOI:** 10.3389/fsurg.2026.1865406

**Published:** 2026-08-21

**Authors:** GuoBin Pan

**Affiliations:** Department of Medical Equipment, Yantaishan Hospital, Yantai, Shandong, China

**Keywords:** artificial intelligence, clinical decision support, foundation models, multimodal deep learning, precision medicine, surgical oncology

## Abstract

Multimodal deep learning is advancing rapidly in surgical oncology, yet fragmented clinical data continue to constrain comprehensive understanding of tumor biology. Here we review progress in artificial intelligence (AI) for precision surgical oncology, with emphasis on its contributions to surgical quality across the preoperative, intraoperative, and postoperative continuum. Preoperatively, radiogenomic models link imaging phenotypes with molecular features to support noninvasive biopsy, neoadjuvant treatment selection, and organ-preservation strategies. Intraoperatively, real-time augmented reality, optical biopsy, and deformable image registration may improve visualization of tumor margins and functional boundaries. Postoperatively, pathomic and multi-omic frameworks may support biology-informed risk stratification and adaptive adjuvant therapy. Critical barriers include limited interpretability, unstable performance under distribution shift, and insufficient prospective evidence. Current evidence supports biologically informed AI as a promising decision-support layer; however, prospective multicenter trials, cost-effectiveness analyses, and governance safeguards are required before routine clinical adoption.

## 1 Introduction

Cancer remains a leading cause of mortality worldwide, and annual incidence is projected to exceed 35 million cases by 2050 (1). Although minimally invasive techniques and enhanced recovery programs have improved perioperative care, surgical decisions still rely heavily on anatomical and morphological assessment. Tumor heterogeneity therefore continues to complicate margin control, recurrence prevention, and functional preservation (2, 3).

Margin assessment in surgical oncology remains predominantly morphological, whereas mutational profiles, proliferative activity, and tumor-microenvironment features are often unavailable until tissue sampling or postoperative analysis. This temporal gap may contribute to missed occult disease or unnecessary removal of functional tissue, motivating decision support that combines anatomy with perioperative biological information (4, 5).

Artificial intelligence can approach expert-level discrimination in selected medical-imaging tasks, but performance may decline after external deployment (6, 7). Data-driven methods are increasingly studied in surgical care (8), although adoption remains slower than in radiology or automated segmentation (9, 10). Unimodal models address defined tasks but may not represent the cross-modal context needed for complex oncological decisions (11).

**Figure 1:**
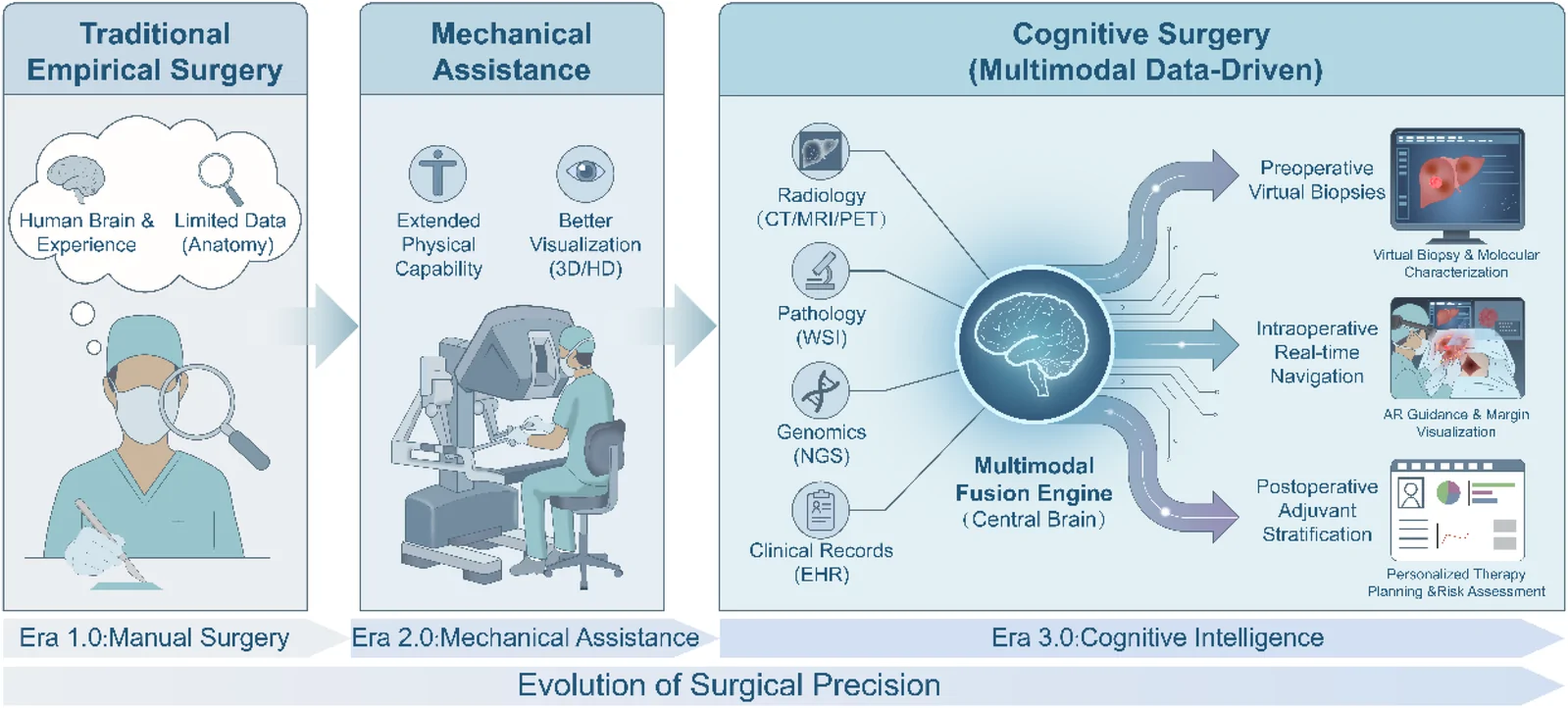
The evolution of surgical precision: From mechanical assistance to cognitive intelligence. Era 1.0 (Manual Surgery): Clinical decisions were based on the surgeon's personal experience and limited anatomical information that was often fragmented. Era 2.0 (Machine-Assisted Surgery): a technological transition driven by robotic surgical platforms and high-definition or three-dimensional visualization systems. Era 3.0 (Cognitive Intelligence): an emerging data-oriented paradigm focused on multimodal information integration. In this framework, a multimodal fusion system combines previously separated clinical data sources, including medical imaging (CT/MRI/PET), pathological whole-slide images (WSI), genomic information from next-generation sequencing (NGS), and electronic health records (EHR).

Multimodal deep learning (MDL) integrates imaging, digital pathology, genomics, and clinical text to identify associations within and across data types (12, 13). These associations may support biomarker discovery, risk stratification, and outcome prediction, but their reliability depends on data quality, cohort representativeness, and external validation (14) (Figure 1).

Fusion may occur at three levels. Early fusion concatenates standardized inputs or features but is sensitive to scale imbalance and missing data. Intermediate fusion uses modality-specific encoders with joint embeddings or cross-attention to model interactions, at greater computational and data cost. Late fusion combines calibrated modality-level predictions, improving modularity and tolerance of missing inputs but potentially losing low-level cross-modal signals. The strategy should be prespecified and tested by modality ablation, calibration, and external validation (13, 14).

This review summarizes MDL methods and perioperative applications in surgical oncology and critically examines their validation, clinical utility, and implementation barriers (15–17).

### 1.1 Literature search and study selection

We conducted a structured narrative search of PubMed/MEDLINE, Web of Science, and Scopus for English-language literature published from January 2017 through January 2026. Search terms combined artificial intelligence, machine learning, deep learning, multimodal learning, foundation model, or large language model with surgical oncology, cancer surgery, perioperative, radiogenomics, augmented reality, digital pathology, or related terms. Reference lists of relevant reviews were also screened.

We included original studies, systematic or narrative reviews, and guidance documents with direct relevance to preoperative, intraoperative, or postoperative surgical oncology. Multicenter, external-validation, prospective, and interventional studies were prioritized. Duplicates, nononcological reports, and purely technical studies without perioperative relevance were excluded. Preprints were retained only for emerging architectures without a peer-reviewed equivalent and are identified as such. Because this was a structured narrative rather than systematic review, publication and selection bias remain possible.

## 2 Preoperative phase: tumor characterization and surgical planning

**Figure 2:**
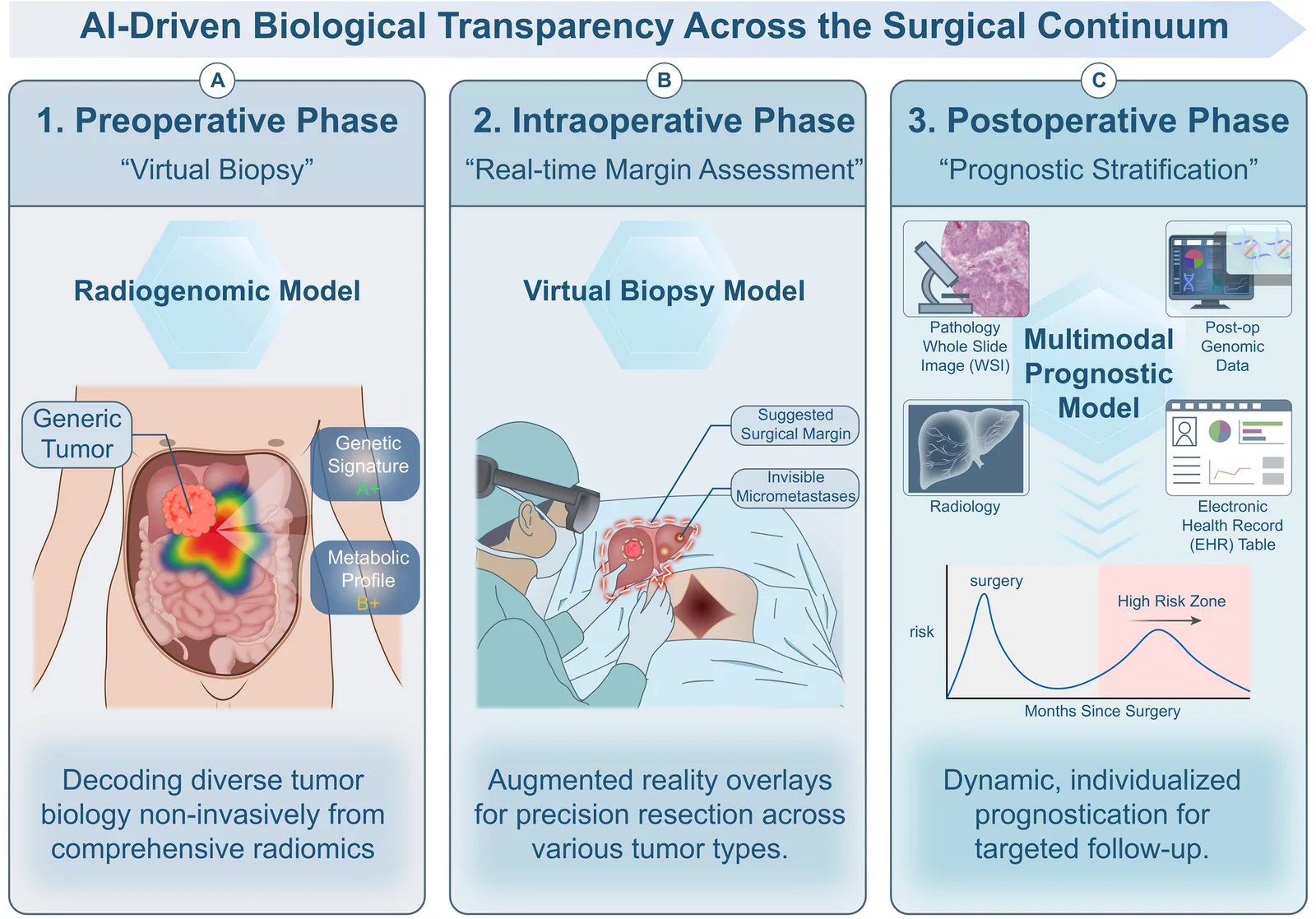
AI-driven biological transparency throughout the surgical process. **(A)** Preoperative phase: Surgeons utilize radiogenomic models to perform noninvasive “virtual biopsies,” characterizing tumor biology prior to surgery by aligning macroscopic imaging information with underlying genetic and metabolic features. **(B)** Intraoperative phase: Real-time assessment of surgical margins is achieved through augmented reality overlays integrated with “virtual biopsy” models. By overlaying predicted margins and micrometastases that conventional methods might miss, AR assists surgeons in achieving precise resections tailored to each tumor type. **(C)** Postoperative phase: Multimodal prognostic stratification. By integrating pathological whole-slide images, imaging findings, postoperative genomic data, and electronic health records, the system delivers dynamic, patient-specific risk predictions to guide targeted follow-up and adjuvant treatment decisions.

Preoperative planning extends beyond resectability to the anticipated margin, operative extent, and sequencing of surgery with neoadjuvant therapy (5, 18). MDL may support these decisions by combining imaging-derived phenotypes with other clinical or molecular data; however, virtual-biopsy outputs should remain adjunctive to established diagnostic pathways (19, 20) (Figure 2A).

### 2.1 Radiogenomics and neoadjuvant therapy guidance

Molecular information can alter preoperative strategy, including mismatch-repair or microsatellite-instability status in colorectal cancer and IDH status in glioma (21). Tissue biopsy remains the reference standard but is invasive and may be affected by intratumoral sampling bias (20, 22). Radiogenomic models seek to estimate spatially distributed molecular features from whole-tumor imaging, but they require tissue-confirmed ground truth and external validation before informing neoadjuvant treatment (5).

#### 2.1.1 Stratification for organ preservation in colorectal cancer

For patients with locally advanced colorectal cancer, pretreatment dMMR/MSI-H status can identify candidates for PD-1 blockade and potential organ-preservation strategies (23). Vision Transformer models have reported external-validation AUCs up to 0.941 for noninvasive MSI prediction (24); however, retrospective design, cohort enrichment, and site-specific imaging or histology may limit transportability. In resource-constrained settings, lighter models using routinely available image features may be more feasible (25), but lower computational cost does not establish clinical utility; local external validation and cost-effectiveness analysis are required before treatment de-escalation.

Functional imaging may add biological context to radiogenomic models (26, 27). In one study, dual-layer spectral CT iodine features were associated with MSI-H status and yielded an AUC of 0.893 (27). Another model combining CT and whole-slide histology reported an AUC of 0.885 (28). These retrospective discrimination metrics do not establish calibration, treatment benefit, or safe selection for organ preservation.

Katsimperis et al. reported cross-study AUCs of 0.80–0.95 for urologic lesion detection, sensitivities and specificities often above 90% for cystoscopic image analysis, and 85%–95% accuracy for renal-cancer mutation prediction (29). These ranges derive mainly from retrospective or single-center studies and are not pooled prospective estimates; limited external validation precludes their use as stand-alone surgical evidence.

#### 2.1.2 Tumor profiling and outcome prediction

MDL frameworks are being investigated for tumor-microenvironment profiling and outcome prediction. An immunophenotype-guided radiomics model inferred immune-cell patterns and reported an AUC of 0.895 for predicting absence of pathological complete response (30). A patho-radiomic model combining CT and histology achieved a concordance index of 0.82 for overall-survival prediction, exceeding TNM staging within the study cohort (31). Both findings are hypothesis-generating; retrospective prognostic discrimination alone should not determine conservative surgery or adjuvant treatment.

#### 2.1.3 Molecular prediction of resection strategy

Radiogenomic models may help characterize advanced tumors before treatment. In gastric cancer, a model stratified patients with occult peritoneal disease into groups with different responses to paclitaxel-based conversion therapy (32), but treatment selection remains unproven prospectively. In glioma, imaging models have predicted IDH1 and TERT alterations associated with biological behavior (21, 33). Such predictions may complement neurosurgical planning, but they should not independently justify extending resection beyond established anatomical and functional boundaries (21, 34).

### 2.2 Detection of occult metastasis and upstaging

Conventional imaging may miss small-volume metastatic disease. Deep-learning radiomics can identify imaging patterns in lymph nodes smaller than 10 mm, but these signals are indirect and require pathology-confirmed validation before they can be interpreted as micrometastases (35).

#### 2.2.1 Preoperative occult metastasis detection for therapeutic sequencing

Accurate mediastinal staging is central to treatment selection in non-small cell lung cancer. PET/CT may be confounded by inflammatory uptake, and suspicious nodes often require invasive confirmation. A DenseNet121 radiomics model combining metabolic and anatomical features showed discrimination between malignant and benign nodes in an external cohort (36). This supports further evaluation as a triage aid, but it does not yet justify omitting guideline-recommended tissue confirmation (36, 37).

Other retrospective models have addressed occult metastasis. In gastric cancer, multimodal prediction of peritoneal metastasis may support selection for staging or conversion therapy (32). In clinically node-negative papillary thyroid carcinoma, a clinical-CT radiomic nomogram predicted occult cervical nodal disease (38), while MRI-based models have been evaluated for preoperative endometrial-cancer staging (39). Prospective decision-impact studies are needed before these predictions alter lymph-node dissection or operative extent.

#### 2.2.2 Defining surgical extent via virtual pathology

When biopsy is hazardous or incomplete, imaging-based models may provide supplementary information but cannot replace histopathology. In pancreatic cancer, automated 3D segmentation can depict the relationship between the tumor and major vessels and may improve consistency of resectability assessment (40). Evidence that this approach reduces futile surgery or improves quality of life remains limited.

Models combining anatomy with histological or molecular subtype may be relevant to retroperitoneal tumors, for which operative strategy varies by biological behavior. Well-differentiated liposarcoma may be managed with a more limited margin in selected cases, whereas dedifferentiated disease can require multivisceral *en bloc* resection. The REMIND framework provides noninvasive subtype predictions, but it remains an adjunct rather than a replacement for multidisciplinary and pathological assessment (41).

## 3 Intraoperative phase: real-time tissue interrogation and navigational guidance

### 3.1 Cross-modal real-time registration and deformation correction

Edge inference can reduce network delay in deformable registration (42, 43), but real-time deployment is constrained by end-to-end latency, not inference alone. A polyp-segmentation benchmark reported more than 180 frames per second (44); this was a technical dataset evaluation rather than evidence of improved patient outcomes. Operating-room use requires synchronized video acquisition, local GPU or edge acceleration, secure high-bandwidth networking, low-latency display, and fail-safe fallback. Studies should report hardware, resolution, throughput, and acquisition-to-display latency because performance may degrade with smoke, occlusion, reflections, and distribution shift (45).

In a prospective usability study of 13 laparoscopic liver procedures, surgeons preferred less visually obstructive overlays, but the displays were not used to guide surgery (46). In a retrospective propensity-weighted cohort (33 AR-assisted and 212 control procedures), conversion for failed tumor localization occurred in 0 vs. 6 cases; however, the difference was not statistically significant, operative time increased by approximately 10%, and resection margins and complications were unchanged (47). These findings support feasibility rather than clinical superiority.

### 3.2 Augmented reality–guided surgical navigation

Augmented reality can project AI-derived spatial information into the operative field, including estimated margins or residual-tumor heat maps. This approach may reduce attention shifts between the operative field and an external monitor, but registration error, visual occlusion, and information overload can introduce new hazards. AR-guided overlays should therefore be treated as navigational aids rather than representations of true microscopic margins (Figure 2B).

The prospective randomized RIDERS trial enrolled 136 patients (133 treated), but only 82 reached 12-month follow-up. AI-driven 3D augmented-reality guidance reduced positive margins at the preserved neurovascular bundle compared with cognitive MRI guidance; however, attrition and short follow-up limit conclusions regarding biochemical recurrence and functional recovery (48). Evidence in neurosurgery is less mature: a retrospective study of 115 patients (39 mixed-reality and 76 standard-navigation procedures) reported smaller residual volumes but no overall-survival benefit, and nonrandom allocation limits causal inference (49).

AR has also been explored in anatomically complex pediatric tumors (50). In liver surgery, structural AR has been combined with indocyanine-green fluorescence to display vascular anatomy and perfusion boundaries (51). These complementary signals may improve visualization, but comparative clinical benefit has not been established. Reported target-registration error was approximately 1.34 mm, and its acceptability depends on the structure at risk, calibration stability, and deformation during surgery (52).

### 3.3 Rapid intraoperative digital pathology and optical imaging

Intraoperative diagnosis requires integration of tissue morphology with clinical and imaging context. Most current task-specific models analyze a single input and therefore cannot independently reproduce this multidisciplinary reasoning; multimodal decision support remains experimental.

#### 3.3.1 Intraoperative diagnostic stratification

Rapid intraoperative diagnosis is most relevant when histology changes the surgical strategy. RapidLymphoma was evaluated prospectively across four centers in 160 patients and in two independent cohorts (*n* = 420 and *n* = 59), achieving balanced accuracies of 97.81%, 95.44%, and 95.57%, respectively, within 3 min (53). Only 25 patients in the prospective cohort had primary CNS lymphoma, and performance at tertiary centers does not establish outcome benefit or transferability to lower-volume settings. A separate single-center study used 126 images from 40 lymphoma patients and therefore provides supporting but less generalizable evidence (54).

#### 3.3.2 Margin assessment and functional preservation

Optical margin assessment remains investigational. Bali et al. trained hyperspectral segmentation models on 712 *ex vivo* datacubes with histopathologic ground truth; the best model achieved 0.98 pixel-level accuracy and 0.93 tumor recall, but the simulated approximately 10-minute workflow was not a prospective intraoperative outcome study (55). In a single-center prospective feasibility study of 22 prostatectomy patients (121 surgical-bed assessments), stimulated Raman histology interpretation achieved 98% accuracy, 83% sensitivity, and 99% specificity and detected 43% of patients with positive margins intraoperatively. The convolutional neural network was tested retrospectively in only 10 consecutive patients; effects on recurrence, function, and cost remain unproven (56).

#### 3.3.3 Managing geometric and depth constraints in optical imaging

Optical imaging is rapid but constrained by irregular tissue geometry and shallow penetration. The rapid arbitrary-shape microscope uses robotic focal adjustment and 3D surface mapping to image unsectioned tissue at subcellular resolution; a proof-of-concept study reported 99.1% sensitivity (57). Photoacoustic spectrum analysis estimates invasion depth from ultrasonic spectral features without exogenous contrast and may assist decisions between endoscopic and radical resection (58). Both approaches remain experimental and require prospective comparison with frozen section or permanent histopathology.

## 4 Postoperative phase: microenvironment profiling and multimodal prognostic stratification

Surgery is followed by risk-adapted surveillance and adjuvant treatment. Resected tissue contains high-dimensional morphological and molecular information that MDL can convert into prognostic features; whether these features improve treatment decisions beyond validated clinicopathological models requires prospective assessment (59) (Figure 2C).

### 4.1 Pathomic-genomic integration for spatial microenvironment profiling

Pathomic-genomic models quantify cell types, spatial distributions, and tissue architecture across whole-slide images (60, 61). In non-small cell lung cancer, spatial profiling has identified patterns associated with resistance to EGFR-targeted therapy, although their ability to select subsequent immunotherapy remains uncertain (62). Efficient-TransUNet automated tumor-stroma-ratio segmentation in colorectal cancer (63). In colorectal liver metastases, an AI-assisted classifier reported AUC above 0.98 for histological growth patterns and increased pathologist accuracy from 75% to 92% in a human-in-the-loop setting (64); multicenter reproducibility and decision impact remain to be established.

A clinically governed human-in-the-loop workflow should display calibrated confidence and an out-of-distribution alert. The surgeon or pathologist should override the recommendation when input quality is inadequate, confidence is below a validated threshold, key modalities are missing, or the output conflicts with anatomy, guidelines, or patient goals. Overrides and subsequent outcomes should be logged for audit, and system failure should default to standard care. The reported increase in diagnostic accuracy from 75% to 92% (64) supports complementarity, not autonomous decision-making, and requires validation across centers and operators.

ETMIL-SSLViT uses self-supervised, transformer-based multitask learning to predict pathological subtype and tumor mutational burden from H&E whole-slide images of endometrial and colorectal cancers (65). A separate colorectal-cancer study integrated multi-omic data and machine learning to investigate associations between Chanling Gao, TP53/CCNB1/CDK1 signaling, and the immune microenvironment (66). These retrospective associations are exploratory and do not establish a causal mechanism or clinical treatment effect.

Gigapixel whole-slide images impose substantial memory and computational demands (67). Mamba-based state-space models and VM-UNet have shown efficient sequence modeling and competitive segmentation on benchmark datasets (68, 69), but benchmark speed does not establish pathology-workflow benefit. Promptable segmentation foundation models adapted to medical images, including MedSAM and the Medical SAM Adapter, have also reported strong benchmark performance (113, 114). TRIP predicted triple-negative breast-cancer status and prognosis from H&E morphology without immunohistochemistry (70), while self-supervised learning can reduce annotation requirements (71). These systems remain research tools until independently validated against current diagnostic and prognostic standards.

### 4.2 Precision adjuvant therapy: strategies for treatment de-escalation and intensification

TNM staging primarily reflects anatomical extent, whereas multimodal models may add biological features associated with prognosis or treatment response (72, 73). Incremental value should be demonstrated through calibration, decision-curve analysis, and prospective comparison with established clinicopathological models.

#### 4.2.1 Biologically-guided treatment de-escalation

The average survival benefit of adjuvant chemotherapy in stage II colorectal cancer is modest and heterogeneous (74). Retrospective AI models have identified subgroups with different prognoses or treatment associations (74, 75), but they do not yet establish who can safely omit chemotherapy. In hormone receptor-positive breast cancer, Orpheus predicted a high Oncotype DX recurrence score with an AUC of 0.89 vs. 0.73 for a clinicopathological nomogram and showed prognostic value among patients with scores of 25 or lower (76). RSClinN + also refined risk estimates in node-positive disease (77). These findings require prospective utility and cost-effectiveness studies before pathology-based models replace genomic assays or direct de-escalation.

#### 4.2.2 Targeted intensification for patients with occult risk

Multimodal AI has also been studied for treatment intensification. In colorectal cancer, retrospective models identified subgroups with hypoxia, immune exclusion, or other high-risk features associated with greater benefit from adjuvant chemotherapy (75, 78). PET/CT radiomics has similarly been associated with response to immune-checkpoint inhibitors in advanced non-small cell lung cancer (79). These associations require prospective predictive validation and should not be interpreted as proof of treatment benefit for an individual patient.

## 5 Large language models in perioperative decision support

### 5.1 LLM-augmented multidisciplinary tumor boards

Retrospective case-based evaluations have reported high concordance between general-purpose LLMs and breast-cancer tumor boards for guideline-concordant scenarios (80, 81). Performance is lower for surgical judgment, genomic-test indications, and individualized risk-benefit trade-offs; models may also overrecommend radiotherapy or differ from experts on chemotherapy and genetic counseling (81, 82). These studies assess recommendation concordance rather than patient outcomes and support supervised decision assistance, not autonomous planning.

In complex pancreatic-cancer scenarios, GPT-4 achieved 61.5% accuracy for neoadjuvant-treatment planning (83). Retrieval-augmented generation can constrain responses to selected sources: an ESMO-guideline system received a median appropriateness score of 9/9 in blinded expert review (84). This evaluation suggests improved guideline grounding but does not establish safety, generalizability, or reduced patient harm.

Disagreement with experts is more common in older patients and those with severe comorbidity (85). Privacy requirements may favor on-premises deployment, which can limit model size, update frequency, or access to external retrieval sources; performance should therefore be validated in the actual local configuration (86). A meta-analysis of 56 studies across 15 cancer types reported overall accuracy of 76.2% and diagnostic accuracy of 67.4%, while most studies did not evaluate patient-safety risks (87).

### 5.2 Vision-language models for intraoperative guidance

LLMs process clinical language, whereas intraoperative support also requires rapid interpretation of changing visual input. Conventional surgical computer vision typically performs predefined tasks such as instrument or phase recognition. Vision-language models (VLMs) extend this approach by linking video or images with text, but current surgical applications are predominantly experimental (88).

Surgical visual-question-answering systems use transformer-based models to answer natural-language questions about operative scenes (89–91). Specialized architectures such as Surgical-MambaLLM and Surgery-R1 aim to improve spatial reasoning and response generation (92, 93); Surgical-MambaLLM is a MICCAI 2025 conference paper, whereas Surgery-R1 remains a preprint, and neither has prospective operating-room validation. Their outputs may be unreliable under occlusion, smoke, bleeding, unfamiliar anatomy, or distribution shift and should not be used as unverified safety alerts.

VLMs may support few-shot surgical-phase recognition and text-based review of steps such as the critical view of safety (91, 94). Text-to-video diffusion models can also generate synthetic operative scenes for research or training (95). Evidence is currently limited to technical datasets and simulated tasks; effects on errors, workload, or patient outcomes have not been demonstrated.

### 5.3 Automated structuring of clinical narratives

Documentation burden can impede surgical quality improvement and translational research. LLMs can convert narrative operative reports into structured variables. In a study of 45 rectal-cancer reports, a HIPAA-compliant framework achieved extraction performance comparable to a surgical resident and reduced processing time from more than two hours to approximately 10 min (96). The small report-level sample and absence of cross-site validation limit generalizability; clinician verification remains necessary.

In a retrospective breast-cancer study, GPT-4 extracted 26 registry variables with 97.1% overall accuracy and reduced median processing time from 15.5 to 1.2 min (97). A multicenter study of GPT-4o for Chinese- and English-language lobectomy records reported accuracy of 0.966 and an F1 score of 0.882 (98). These results support assisted data curation, but field-level error analysis, privacy controls, and human quality assurance remain essential.

**Figure 3:**
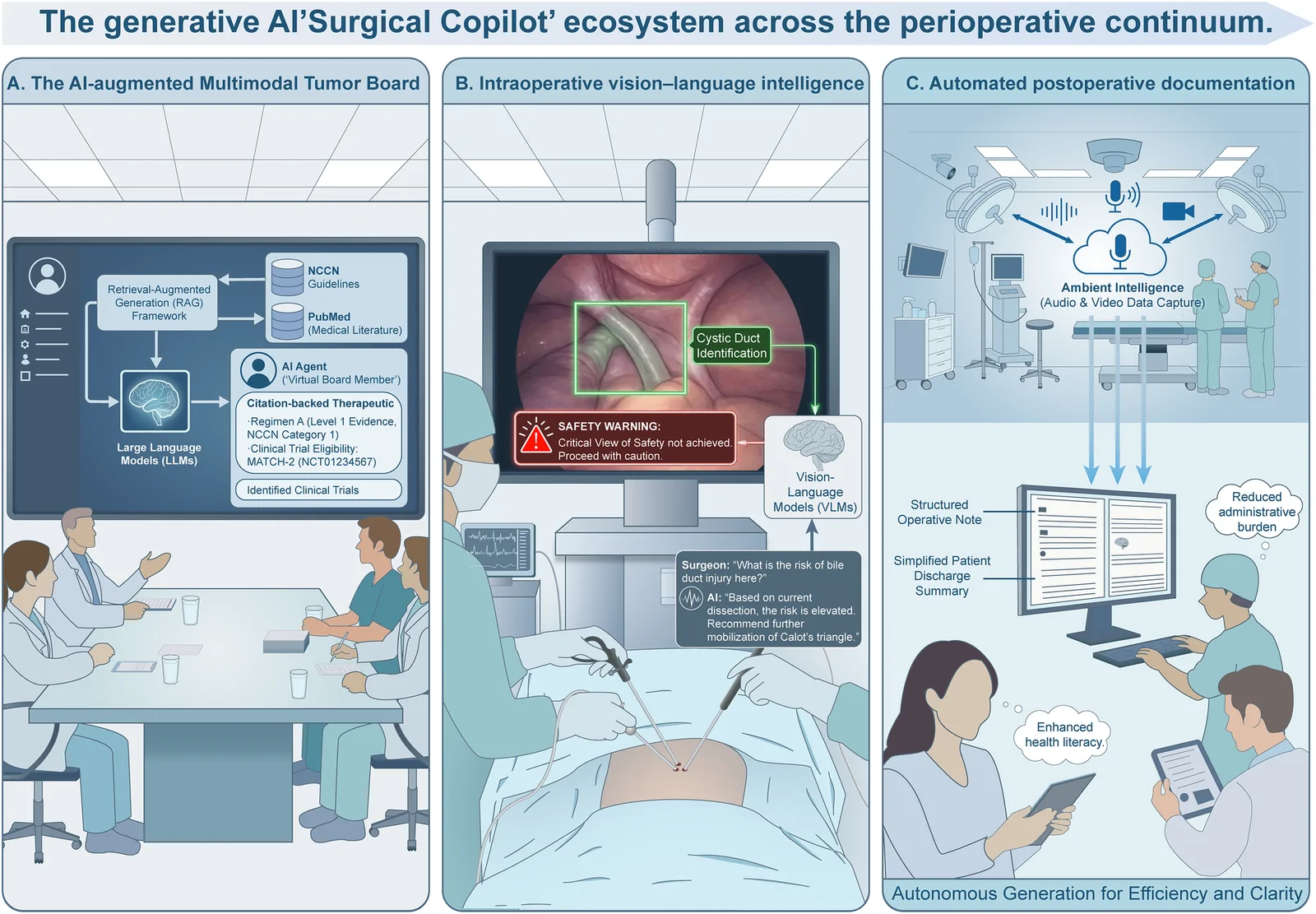
The generative AI-driven intelligent support ecosystem across the perioperative Continuum. **(A)** AI-Enhanced Multimodal Tumor Board: LLMs using a Retrieval-Augmented Generation (RAG) framework act as intelligent clinical decision-support tools. LLMs reference established guidelines and literature to support therapeutic recommendations and help identify relevant clinical trials, with mechanisms in place to reduce erroneous suggestions. **(B)** Intraoperative Vision-Language Intelligence: VLMs deliver proactive safety alerts and dynamic risk evaluations through natural language interactions during surgical procedures. **(C)** Automated Postoperative Documentation: Ambient intelligence platforms collect audio and video intraoperative data to automatically generate structured operative reports and patient-friendly discharge summaries.

Generative AI has also been evaluated for rewriting operative notes in patient-friendly language. Studies in gynecological oncology and otorhinolaryngology reported improved readability while maintaining high expert-rated medical accuracy (98, 99). Because simplification can omit uncertainty or alter meaning, generated discharge instructions require clinician review and patient testing before routine use (Figure 3C).

For postoperative breast-cancer recommendations, a guideline-augmented model achieved an accuracy of 0.89, compared with 0.86 for Claude-2 and 0.78 for GPT-4 (100). In 98 complex oncology cases, guideline concordance was 92%–96%, but agreement with experts was only 68.4%–76.5% (101). The gap likely reflects patient-specific factors not fully represented in prompts. LLM output should therefore be treated as reviewable advice, with accountable clinicians retaining final decision authority.

LLMs can derive predictive features from unstructured perioperative notes. One study reported absolute AUROC and AUPRC improvements of 38.3% and 33.2%, respectively, over traditional word embeddings for postoperative-risk prediction (102). Other studies report accuracy above 90% for selected oncology extraction tasks (103). These task-specific results require external validation, calibration, prospective workflow evaluation, and monitoring for subgroup performance before clinical risk alerts are deployed.

## 6 Challenges to clinical translation and future directions

AI applications in surgical oncology remain at different stages of evaluation, from retrospective radiogenomic models and preclinical optical imaging to prospective diagnostic studies and early randomized navigation trials. Experimental performance should be distinguished from clinical validation, and clinical validation from demonstrated utility or regulatory approval. Major risks include biased training cohorts, weak external validation, automation-related patient harm, privacy or cybersecurity breaches, opaque reasoning, and uncertain responsibility when errors occur.

Technical discrimination should not be equated with clinical utility. Most studies reviewed are retrospective or single-center and report internal AUC or accuracy rather than calibration, treatment change, safety, or patient outcomes. Clinical readiness requires independent external validation, prospective silent testing, and then interventional evaluation with predefined failure modes, subgroup analyses, clinician-AI interaction, and workflow or patient outcomes (104) (Table 1).

**Table 1:** Evidence maturity of representative perioperative AI applications.

Application (references)	Evidence base and validation	Evidence stage	Main limitation
Colorectal MSI prediction (24–28)	Mostly retrospective cohorts; some external validation; reported AUC 0.885–0.941.	Retrospective external validation	Site-specific imaging and selection bias; no treatment-impact trial.
Occult metastasis and virtual pathology (32, 36–41)	Predominantly retrospective model development and validation.	Experimental decision support	No evidence that use safely avoids biopsy or improves outcomes.
AR liver navigation (46, 47)	Prospective usability study (13 procedures) and retrospective comparison (33 AR vs. 212 controls).	Prospective feasibility	No significant margin or complication benefit; longer operating time.
AR prostate navigation: RIDERS (48)	Randomized trial: 136 enrolled, 133 treated, 82 assessed at 12 months.	Early randomized clinical evaluation	Attrition and short follow-up; recurrence and functional benefit remain uncertain.
RapidLymphoma/SRH (53, 54)	Prospective four-center cohort (*n* = 160), independent cohorts (*n* = 420 and *n* = 59), plus a smaller single-center series.	Prospective multicenter diagnostic validation	Few primary CNS lymphoma cases; no demonstrated outcome or cost benefit.
Optical margin assessment (55, 56)	*Ex vivo* hyperspectral data (712 datacubes); prospective feasibility in 22 patients; CNN tested retrospectively in 10 patients.	Experimental/feasibility	Small, single-center or *ex vivo* studies; recurrence and functional outcomes untested.
Stage II colorectal risk models (74, 75)	Multicenter retrospective development with external validation; one study included 2,992 patients.	Retrospective multicenter validation	Predictive treatment benefit has not been established in a randomized trial.
Breast pathomic risk prediction (76)	Retrospective three-institution cohort of 6,172 cases.	Retrospective multicenter validation	Uses recurrence score and retrospective outcomes; cannot replace genomic testing.
LLM tumor boards and documentation (80–103)	Mostly retrospective case vignettes or report-level evaluations; operative-report study included 45 reports.	Experimental decision support	Variable expert concordance, hallucination, privacy risk, and no patient-outcome trial.

Validation stage describes the cited study design, not regulatory approval or established patient benefit. None of these tools supports autonomous clinical use.

### 6.1 Model interpretability and interactive explainability

Clinical AI requires evidence that clinicians can inspect and challenge. Explainable-AI methods such as attention maps can show which input regions influenced a model but do not necessarily reveal causal reasoning or guarantee correctness. Explanations should therefore be evaluated for fidelity, stability, and usefulness in the intended workflow rather than assumed to confer trust (80, 82, 99, 100).

Interactive explainable AI allows clinicians to query a model through a dialogue interface and may retrieve similar cases or display calibrated confidence (84, 105, 106). Such interfaces can improve usability, but fluent explanations may still be *post hoc* or incorrect. Their evaluation should include failure detection, clinician reliance, override behavior, and effects on decisions.

Because errors may arise from model design, local integration, data shift, or clinician use, responsibility may be distributed among developers, institutions, and clinicians. Deployment therefore requires versioned audit logs, defined oversight, incident review, and local medico-legal assessment; liability standards remain unsettled, so AI output should remain advisory rather than replace accountable clinical judgment (107).

### 6.2 Algorithmic robustness to data sparsity and missing modalities

Routine clinical data are incomplete and heterogeneous. Genomic sequencing may be unavailable because of cost, imaging protocols vary across institutions, and intraoperative tissue may be limited (4, 5). Models trained on complete academic datasets can therefore fail when a modality is missing or has shifted distribution (108). Reported performance on curated cohorts should not be described as robust without explicit missing-data and external-site testing.

Cross-modal imputation uses available low-cost modalities to estimate missing high-dimensional inputs, while modality dropout trains a model to operate with variable inputs (28, 76, 112). These methods may improve resilience but can propagate systematic error and should expose uncertainty rather than silently synthesize unavailable data. Federated and swarm learning enable distributed model development without centralizing raw records, but privacy leakage, site imbalance, and protocol heterogeneity remain concerns (109, 110). LoRA reduces the number of trainable parameters required to adapt a foundation model; it does not by itself ground outputs in clinical variables or ensure clinical validity (111).

Access remains unequal: genomic sequencing, whole-slide scanners, stimulated Raman histology or augmented-reality systems, and on-premises accelerators require capital, maintenance, annotation expertise, and cybersecurity support. Tiered deployment should compare incremental clinical benefit with total cost; simpler image-only or late-fusion tools may be more feasible but provide narrower biological coverage. Standardized platforms such as PIONEER and federated or swarm learning may reduce data silos without centralizing raw data, but they do not remove selection bias, protocol heterogeneity, or the need for external validation (29, 109, 110).

## 7 Conclusion

Multimodal deep learning and foundation models may improve patient-specific risk stratification by integrating imaging, pathology, genomics, and clinical data. However, most evidence remains retrospective or task-specific, and routine adoption requires multicenter prospective validation, cost-effectiveness analysis, robust handling of missing data and distribution shift, and clear governance. Clinically useful systems should augment, rather than replace, surgeons through transparent uncertainty, override pathways, and auditable accountability.
